# Gated Attention-Augmented Double U-Net for White Blood Cell Segmentation

**DOI:** 10.3390/jimaging11110386

**Published:** 2025-11-01

**Authors:** Ilyes Benaissa, Athmane Zitouni, Salim Sbaa, Nizamettin Aydin, Ahmed Chaouki Megherbi, Abdellah Zakaria Sellam, Abdelmalik Taleb-Ahmed, Cosimo Distante

**Affiliations:** 1 Laboratory of Vision Systems and Communication (VSC), Department of Electrical Engineering, University of Mohamed Khider Biskra, Biskra 07000, Algeria; ilyes.benaissa@univ-biskra.dz (I.B.); athmane.zitouni@univ-biskra.dz (A.Z.);; 2 Computer Engineering Department, Faculty of Computer and Informatics, Istanbul Technical University, Istanbul 34485, Turkey; naydin@itu.edu.tr; 3 Laboratory of Identification, Command, Control and Communication (LI3C), Department of Electrical Engineering, University of Mohamed Khider, Biskra 07000, Algeria; 4 Department of Innovation Engineering, University of Salento, 73100 Lecce, Italy; 5 Institute of Applied Sciences and Intelligent Systems, Consiglio Nazionale delle Ricerche (CNR), 73100 Lecce, Italy; 6 Laboratory of Institute of Electronics, Microelectronics and Nanotechnology (IEMN), UMR CNRS 8520, Université Polytechnique Hauts-de-France, 59309 Valenciennes, France

**Keywords:** medicalimaging, white blood cell image segmentation, supervised deep learning, convolutional neural networks, attention-augmented convolution, gating mechanism

## Abstract

Segmentation of white blood cells is critical for a wide range of applications. It aims to identify and isolate individual white blood cells from medical images, enabling accurate diagnosis and monitoring of diseases. In the last decade, many researchers have focused on this task using U-Net, one of the most used deep learning architectures. To further enhance segmentation accuracy and robustness, recent advances have explored the combination of U-Net with other techniques, such as attention mechanisms and aggregation techniques. However, a common challenge in white blood cell image segmentation is the similarity between the cells’ cytoplasm and other surrounding blood components, which often leads to inaccurate or incomplete segmentation due to difficulties in distinguishing low-contrast or subtle boundaries, leaving a significant gap for improvement. In this paper, we propose GAAD-U-Net, a novel architecture that integrates attention-augmented convolutions to better capture ambiguous boundaries and complex structures such as overlapping cells and low-contrast regions, followed by a gating mechanism to further suppress irrelevant feature information. These two key components are integrated in the Double U-Net base architecture. Our model achieves state-of-the-art performance on white blood cell benchmark datasets, with a 3.4% Dice score coefficient (DSC) improvement specifically on the SegPC-2021 dataset. The proposed model achieves superior performance as measured by mean the intersection over union (IoU) and DSC, with notably strong segmentation performance even for difficult images.

## 1. Introduction

White blood cell (WBC) segmentation is a critical step for the automated classification and AI-based diagnosis of blood cancers. Automating this process overcomes the slow and labor-intensive nature of manual methods, enabling faster, more consistent, and large-scale analysis [1,2,3]. Advances in big data and deep learning have accelerated the development and widespread implementation of clinical decision support systems in healthcare [4,5]. However, inconsistencies in imaging protocols and equipment compromise analytical validity, prompting early traditional methods to address these via color adjustment techniques, such as transferring color statistics from a template image in lab space followed by color space decomposition and K-means clustering for nucleus and cytoplasm segmentation [6]. This underscores the need for machine learning techniques that are robust to such variability [7].

Research using microscopic imaging allows for the visualization and analysis of different blood cell types: white blood cells, red blood cells, and platelets. WBCs have five types: neutrophils, eosinophils, monocytes, basophils, and lymphocytes. Each contains a nucleus and cytoplasm [8,9,10]. Identifying and classifying WBCs are crucial for cell counts and early diagnosis of various diseases, including infections, leukemias, and immune disorders [11,12,13,14]. Machine learning and deep learning are increasingly used with traditional methods for image segmentation and are classified as supervised, weakly supervised, or unsupervised based on the availability of labeled data [15,16,17]. The unsupervised learning process is more complex since it lacks labeled data. On the other hand, weakly supervised learning requires only a small portion of labeled data, while the majority remain unlabeled. Unlike unsupervised and weakly supervised learning, supervised learning requires that all data be carefully labeled, usually by experts, to ensure the credibility and accurate training of models [18].

Various supervised methods are commonly used to solve the biomedical segmentation problem. One of the most used methods in this field is U-Net [19], an auto-encoder that uses skip connections between encoder and decoder blocks to retain important fine-grained features that may be lost during the downsampling and upsampling processes [20]. The skip-connected encoder-decoder design of U-Net makes it a core framework in most medical image segmentation tasks [21]. Despite this advantage, its performance can be limited in handling complex structures or variations in an image, such as small, intricate features or varying resolutions. This has led researchers to develop variations of the U-Net architecture, such as Nested U-Net (or U-Net++) [21], which incorporates nested, dense skip connections to improve feature fusion and better capture fine-grained details, and Double U-Net [22], which consists of two encoders and two decoders. The first encoder utilizes the VGG19 architecture, while the second encoder adopts a structure similar to that of the U-Net encoder, with its decoder connected to the first path by an additional long skip connection to allow features from the first encoder to directly contribute to the second decoder’s outputs [23]. These techniques still face challenges in acquiring enhanced global representations for accurately defining the specific WBC positions and boundaries [24], which can restrict the performance of segmentation. Recent studies have focused more on attention-based U-Net [25], creating a wide range of U-Net-like varieties such as Attention-Augmented U-Net (AA-U-Net) [26] to combine the strength of U-Net and capture both local and long-range features by virtue of the attention mechanism [27,28,29].

In this paper, we propose an enhanced double encoder-decoder architecture for WBC segmentation, termed Gated Attention-Augmented Double U-Net (GAAD-U-Net), which is modified from the baseline Double U-Net architecture [22]. To deal with the encoder path of our network, we employ a tailored strategy that reduces both the parameter count and computational complexity while preserving key semantic information in the early layers. This approach helps integrate long-range spatial cues into the low-level feature maps. The robust features obtained from these initial layers are then passed through custom-built gated attention-augmented convolution blocks, which amplify critical feature representations through a multi-path attention mechanism. Each path utilizes a distinct series of convolutional operations, enabling the network to capture multi-scale receptive fields more effectively.

Our Double U-Net framework incorporates skip connections to mitigate potential gradient vanishing issues as the model deepens. In the decoder stage, the encoded feature maps at each downsampling layer are combined via skip connections with the corresponding upsampled output. The same gated attention-augmented blocks are subsequently applied to refine these fused features, enhancing the overall segmentation performance. Notably, GAAD-U-Net does not require ancillary data, such as pre-initialized masks or boundary supervision, making it straightforward to train the model in an end-to-end manner. We summarize our main contributions in this work as follows:

- Attention-augmented convolutions are integrated to selectively emphasize informative features across channels and spatial scales.
- Gating mechanisms are incorporated to suppress irrelevant regions and enhance focus on meaningful anatomical structures.
- Unlike recent studies, this method was tested on both individual WBCs (cropped cell images) or multiple cells within one image. Our approach handles both scenarios effectively.
- The proposed method achieves accurate and efficient segmentation without relying on additional support inputs or specialized preprocesses.

## 2. Related Work

Recent advancements in segmentation methods help identify the WBCs within blood images, since they distinguish each cell’s features from the background. This process will later ease accurate cell counting, morphological analysis, and blood disease diagnosis. Within this section, a literature overview of existing computer vision algorithms used for this task is presented and divided into two main approaches: (1) convolutional neural networks and (2) attention mechanism-integrated networks.

**(1) Convolutional Neural Networks**

In order to solve WBC issues, the Lu et al. [30] approach integrates ResNet as a backbone for the Nested U-Net architecture to enhance feature extraction. They tested their methodology on four WBC datasets and reported an average micro IoU of 0.96. However, according to [31], they did not compare their methodology with other deep semantic segmentation networks. In the work presented by Roy and Ameer [32], it can be observed that the authors implemented the architecture of DeepLabv3+, which is essentially based on deep semantic understanding. The ResNet-50 network was applied to this model as its backbone to extract features from images. The experimental settings were three different public datasets. These approaches led to a high mean IoU of 0.921. However, the study had a shortcoming in terms of the specific detail relating to the efficiency of the model in segmenting nuclear components. Guo et al. [31] integrated the concept of indeterminacy from neutrosophic WBCs sets on widely recognized CNNs to ensure robustness in varying image conditions, such as the brightness and resolution, obtaining 0.90887 and 0.82069 for the mean IoU and mean BFScore, respectively, on the SegPC-2021 dataset. Zhang et al. [33] proposed RotU-Net, an encoder-decoder U-Net-like architecture. The key change in this paper is leveraging the weight rotator block alongside the feature expansion module and feature restoration module as a bottleneck for the original U-Net architecture, achieving a 0.8201 DSC on the SegPC dataset.

**(2) Attention Mechanism-Based Networks**

The Chen Li et al. [34] approach involved a proposal of an attention-based nested U-Net set-up, which segments white blood cells by inserting attention gates into the traditional Nested U-Net to help extract the features in each layer of the network. It achieved an IoU of 0.8017 on a small WBC dataset. Dongming Li et al. [35] proposed an architecture called CBAM-DC-UNet that combines both a convolutional block attention module (CBAM) and dilated convolution (DC) in the traditional U-Net design. The authors replaced the original RMSProp optimizer with the RAdam optimizer, which adapts to the learning rate efficiency. This showed great efficiency when dealing with complex scenarios, like adherent cells and ambiguous boundaries. However, the segmentation was not performed on the nucleus and cytoplasm separately. Instead, it focused more on segmenting the WBC instance as a single class. Liu et al. [36] suggested a robust model named DCSAU-Net specifically developed for medical image segmentation, including WBC segmentation. This model enhances the U-Net design by integrating a split-attention block that facilitates multi-dimensional attention across various channel dimensions. Recent advancements underscore the value of multi-scale channel-spatial attention for preserving intricate details in medical imaging, as reviewed in super-resolution contexts for endoscopic tasks [37], where integrated channel and spatial modules (e.g., in CCSBESR) enhance edge fidelity via parallax-aware refinement. Our GAAD-U-Net set-up extends this paradigm by embedding AAC and SE blocks within a Double U-Net framework, enabling superior multi-scale boundary detection in WBC segmentation without ancillary data. Extensive experiments have further proven its exceptional performance in segmentation tasks and yielded a mean IoU of 0.806 and a 0.886 DSC. GA2Net [38] is another robust model that uses hierarchical gated feature aggregation, adaptive skip connections, and mask-guided attention mechanisms in the decoder while incorporating deep supervision at multiple stages to better delineate tissue boundaries across diverse medical imaging tasks, achieving a 0.9274 DSC. However, this method was not fully automated in the WBC segmentation task and relied on preprocessed, cropped WBC images, limiting its practical applicability in fully automated blood smear analysis.

Table 1 shows a concise overview of key architectural distinctions across other models. Both GAAD-U-Net and AA-U-Net utilize the same foundational AAC block, but their approaches differ markedly. AA-U-Net applies a single global context injection at the bottleneck of a standard U-Net set-up, while GAAD-U-Net employs a dual-phase Double U-Net architecture, deploying modules across both stages and replacing the bottleneck with a sequential refinement cascade (AAC → Gating → ASPP) for enhanced feature analysis. Its dual-phase skip connections also provide a more sophisticated fusion than AA-U-Net’s basic connections. Comparatively, GAAD-U-Net uses spatial self-attention via AAC to capture long-range pixel dependencies, unlike DCSAU-Net’s channel-wise attention via CSA for feature recalibration. Architecturally, GAAD-U-Net focuses on a two-stage, coarse-to-fine process with a complex bottleneck, while DCSAU-Net emphasizes efficient multi-scale extraction in a single-pass U-Net framework.

Despite the superiority of these latest methods, it is worth mentioning three common cons that have been widely arising in the state of the art:

**(1) Performance in complex cases:** While state-of-the-art models perform extremely well in most cases, they may struggle with complex scenarios, such as close boundaries or overlapping cells. This creates a research gap where new methods need to be developed to enhance the performance of these models in broader scenarios.

**(2) Evaluating performance for WBCs as a single component:** Most state-of-the-art models segment WBCs as a single component, which limits detailed analysis by evaluating the segmentation performance of the cytoplasm and nucleus separately. By treating the cytoplasm and nucleus as separate entities, we can capture subtle variations in their shapes and textures, leading to improved segmentation performance and more reliable results in medical imaging applications.

**(3) Adhesion issues:** Most existing studies focus on segmenting isolated WBCs in images, neglecting the common real-world scenario where WBCs adhere to or overlap with other blood cells such as red blood cells and blood platelets. This limitation leads to models that perform well in controlled settings but struggle in complex cases with cell clusters.

While state-of-the-art techniques in WBC segmentation have shown promising results, several limitations persist that hinder their widespread adoption and performance in diverse scenarios. Many methods struggle to generalize to diverse datasets with varying resolutions, lighting conditions, and staining techniques, reducing their robustness in real-world applications. Additionally, most approaches segment WBCs as a single entity, failing to distinguish between cytoplasm and nucleus, which limits their utility in capturing the subtle morphological details crucial for disease diagnosis. Advanced models often come with high computational complexity, requiring significant hardware resources and making them less practical for deployment in resource-constrained settings. Furthermore, challenges like overlapping cells, noisy images, and ambiguous boundaries continue to impact accuracy, while reliance on small, curated datasets exacerbates issues like overfitting. The tradeoff between accuracy and speed and the limited interpretability of complex models further complicate their integration into clinical workflows, highlighting the need for more robust, efficient, and interpretable solutions.

To address these limitations, we present GAAD-U-Net, a novel approach to WBC segmentation that leverages the power of attention-augmented convolution to enhance the performance of the Double U-Net architecture. By incorporating attention mechanisms, GAAD-U-Net focuses on the most relevant features within the cytoplasm and nucleus, enabling precise segmentation of these critical components even in challenging scenarios such as overlapping cells or ambiguous boundaries. The integration of attention-augmented layers ensures the model captures both the global context and fine-grained details, overcoming issues like poor contrast and morphological variability. Additionally, the Double U-Net framework improves hierarchical feature extraction, allowing for better generalization across diverse datasets. GAAD-U-Net is designed to balance high segmentation accuracy with computational efficiency, making it suitable not only for achieving state-of-the-art performance but also for real-world deployment in clinical applications. Through these innovations, GAAD-U-Net addresses the key challenges faced by existing methods and takes a significant step toward more reliable and interpretable WBC segmentation.

## 3. Method

**Figure 1:**
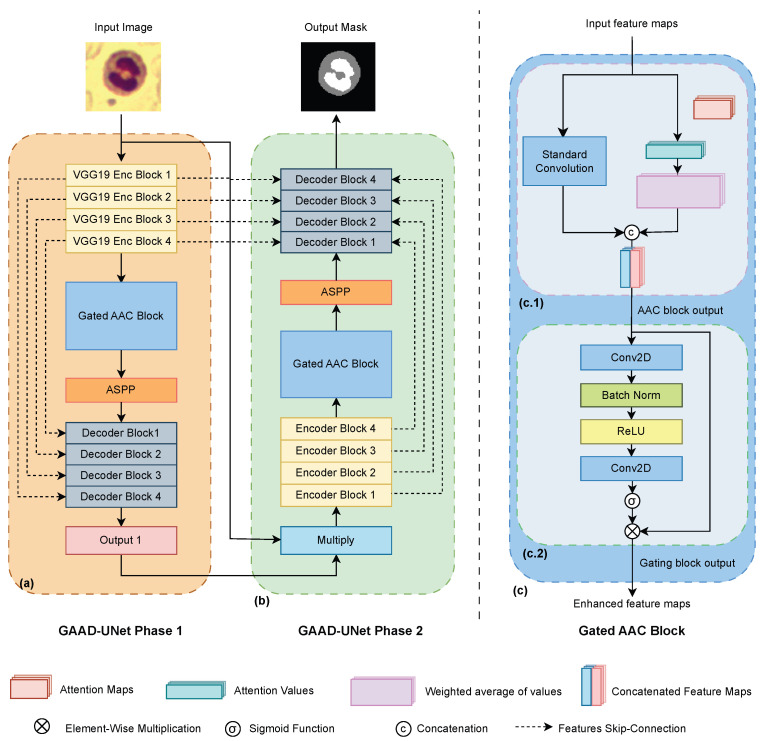
Overview of the proposed GAAD-U-Net architecture for white blood cell segmentation. (**a**) Phase 1 utilizes a VGG19 encoder, a Gated AAC block, an ASPP module, and a decoder to generate the initial segmentation. (**b**) Phase 2 refines this output using a second encoder-decoder pipeline. (**c**) The Gated AAC block, detailed in (**c.1**,**c.2**), represents attention and gating mechanisms, respectively.

We propose a dual-path Double-U-Net architecture that incorporates a gated attention-augmented convolution (Gated AAC) module within its bottleneck. This design, as shown in Figure 1, facilitates tailored feature extraction by dedicating separate processing streams to the nucleus and cytoplasm components, thereby enhancing sensitivity to fine morphological details. The Gated AAC module leverages attention to focus on salient features and a gating mechanism to refine representations, while the cascaded U-Net structure captures multi-scale context. This approach yields distinct, component-specific segmentations critical for advancing automated hematological analysis.

### 3.1. Background Knowledge

#### 3.1.1. Double U-Net Baseline

WBC images are microscopic in nature and contain highly detailed structures, including cell boundaries, nuclei, and tissue textures, which can be challenging to capture in a single pass. Additionally, annotated datasets for white blood cell segmentation are often limited due to the high cost and expertise required for manual labeling. To address these challenges, we employ a two-stage U-Net-based architecture, where the first U-Net generates a coarse segmentation and the second U-Net refines details by improving boundary delineation and reducing false positives through error correction from the first stage. This sequential refinement process enhances segmentation performance, particularly in scenarios with limited training data, by enabling better feature learning and reducing overfitting.

#### 3.1.2. Attention-Augmented Convolution (AAC) Block

The attention-augmented convolution (AAC) block shown in Figure 1(c.1) enhances standard convolutional operations by integrating self-attention mechanisms, combining features extracted via convolution with those obtained through self-attention mechanisms and enabling the model to capture both local and global spatial dependencies. Given an input tensor $x\in R^{B\times C\times H\times W}$, where *B* is the batch size, *C* is the number of input channels, and H×W are the spatial dimensions, the AAC block is formulated as

(1) $$y_{\mathrm{aac}}=\mathrm{Concat}\left(\mathrm{Conv}2D(x),\mathrm{Attention}(x)\right),$$

where the following definitions apply:

- Conv2D(x) is a standard 2D convolution on the input tensor *x*, producing a feature map $y_{\mathrm{conv}}\in R^{B\times C_{\mathrm{conv}}\times H\times W}$, where $C_{\mathrm{conv}}$ is the number of output channels for the convolutional branch.
- Attention(x) is the self-attention on *x* that calculates attention weights to obtain global spatial relationships. The attention module produces a feature map $y_{\mathrm{attn}}\in R^{B\times C_{\mathrm{attn}}\times H\times W}$, where $C_{\mathrm{attn}}$ is the number of output channels of the attention branch.
- Concat(·) concatenates the output of the attention and convolution branches along the channel axis to produce the final output $y_{\mathrm{aac}}\in R^{B\times (C_{\mathrm{conv}}+C_{\mathrm{attn}})\times H\times W}$.

The self-attention mechanism of the AAC block is calculated as follows:

(2) $$\mathrm{Attention}\left(x\right)=\mathrm{Softmax}\left(\frac{QK^{T}}{\sqrt{d_{k}}}\right)V,$$

where the following definitions apply:

- Q,K,V are the query, key, and value matrices, respectively, obtained from linear transformations of the input tensor *x*. These matrices are computed as
  $$Q=W_{Q}.x,\;K=W_{K}.x,\;V=W_{V}.x,$$
  where $W_{Q},W_{K},W_{V}$ are learnable weight matrices ($W_{Q}$ is the query projection matrix, $W_{K}$ is the key projection matrix, and $W_{V}$ is the value projection matrix).
- The dimensionality of the key vectors is $d_{k}$, which is used to scale the dot product attention scores and prevent vanishing gradients.
- Softmax(·) normalizes the attention scores to produce a probability distribution over the spatial locations.

Inspired by Patil et al. [39], who successfully integrated the AAC module into the conventional U-Net architecture, we extended this approach to our dual-phase framework. Given that U-Net serves as the baseline for most modern medical segmentation architectures, we recognized that AAC blocks offer significant potential beyond their traditional applications in classification and object detection, specifically for biomedical segmentation tasks.

The integration of an attention-augmented convolution (AAC) block within a network bottleneck enables the simultaneous capture of both local and global feature representations. For microscopic image analysis, such as WBC segmentation, this dual functionality is critical; the AAC block captures fine-grained details like cell boundaries and nuclei while concurrently modeling the overall cellular morphology. This synthesis of convolutional and self-attention mechanisms facilitates a more exhaustive feature extraction, thereby improving segmentation accuracy.

Furthermore, the bottleneck design enhances computational efficiency by minimizing parameters and computations without degrading performance. By optimizing the feature representation to emphasize the most salient information, this integration establishes a powerful and efficient framework for feature extraction in complex biomedical imaging tasks.

#### 3.1.3. Gating Mechanism

As presented Figure 1(c.2), the gating block applies a gating mechanism to an input tensor $x\in R^{B\times C\times H\times W}$, where *B* is the batch size, *C* is the number of input channels, and H×W are the spatial dimensions. The formula for the gating mechanism is defined by

(3) $$z=x⊙\sigma \left({\mathrm{Conv}2D}_{2}\left(\mathrm{ReLU}\left(\mathrm{BatchNorm}\left({\mathrm{Conv}2D}_{1}\left(x\right)\right)\right)\right)\right),$$

where the following properties apply:

- ${\mathrm{Conv}2D}_{1}$ is a 1 × 1 convolution that reduces the number of channels from *C* to C/2, producing an intermediate feature map $y_{1}\in R^{B\times C/2\times H\times W}$.
- BatchNorm applies batch normalization to $y_{1}$, resulting in $y_{2}\in R^{B\times C/2\times H\times W}$.
- ReLU applies the rectified linear unit activation function to $y_{2}$, producing $y_{3}\in R^{B\times C/2\times H\times W}$.
- ${\mathrm{Conv}2D}_{2}$ is a 1 × 1 convolution that expands the number of channels back from C/2 to *C*, yielding $y_{4}\in R^{B\times C\times H\times W}$.
- The sigmoid function is denoted by σ, which generates the gating mask $g\in R^{B\times C\times H\times W}$.
- Element-wise (Hadamard) multiplication is signified by ⊙, scaling the input *x* with the gating mask *g* to produce the final output $z\in R^{B\times C\times H\times W}$.

The gating mechanism is added to enhance the model’s ability to focus on salient features while disregarding less important ones, thereby improving feature selection and representation learning. In the context of microscopic and WBC images, where fine-grained details and subtle variations are crucial, the gating mechanism enables the network to prioritize essential structures, such as cell boundaries or nuclei, while mitigating noise.

When employed after attention-augmented convolution, the gating mechanism further refines the feature maps by integrating the global context captured by attention with localized feature selection. This is particularly beneficial for WBC images, as identifying cell types or anomalies requires both the global context and detailed local textures. The blend of attention and gating enhances feature extraction.

### 3.2. Architecture

The GAAD-U-Net architecture, depicted in Figure 1, represents a significant enhancement of the conventional Double U-Net model for white blood cell segmentation. This novel design incorporates two distinct phases, each representing a U-Net network, optimized for feature extraction and segmentation refinement.

The key change in our architecture lies in integrating a Gated AAC module in the bottlenecks of the Double U-Net model. This method is particularly beneficial in medical imaging tasks, where understanding relationships across the entire image can significantly improve performance.

Table A1 outlines the block-wise specifics of the GAAD-U-Net architecture with the output shape of each block and its trainable parameters.

Table A3a highlights the configuration parameters of the AAC block used in our architecture. This block combines standard convolution with self-attention mechanisms, utilizing 512 input and output channels with a kernel size of three. The attention mechanism is configured with 32-dimensional keys and values distributed across four attention heads. It implements relative positional encoding (relative = true) and operates at a stride of one on feature maps with a spatial dimension of 28 × 28. This architecture allows the block to capture both local spatial relationships through convolution and long-range dependencies through multi-headed self-attention.

#### 3.2.1. GAAD-U-Net First Phase

The first phase of GAAD-U-Net, shown in Figure 1a, employs the first four VGG-19 encoder blocks, where AAC modules are strategically integrated into the bottleneck of this VGG-19 set-up. This AAC block enhances the network’s ability to focus on salient features critical for accurate cell segmentation. Following the AAC block, a gating block is used to improve the focus on relevant anatomical structures while suppressing irrelevant regions and mitigating semantic gaps, which reduces false positives in segmentation. An Atrous Spatial Pyramid Pooling (ASPP) module is applied after the gated AAC to capture multiscale contextual information.

The decoder section of Phase 1 (left) comprises four sequentially arranged decoder blocks, each implementing upsampling and feature refinement. Each Phase 1 decoder block performs bilinear upsampling to double the spatial dimensions, concatenates with the corresponding encoder skip connections to preserve fine-grained details, and then processes the merged features through successive convolutions followed by a squeeze-and-excitation (SE) layer to enhance channel-wise feature extraction and recalibration. This design ensures optimal spatial resolution recovery while maintaining focus on discriminative features during the reconstruction process, effectively combining local detail preservation with semantic feature refinement for accurate segmentation output.

#### 3.2.2. GAAD-U-Net Second Phase

Phase 2 (right), illustrated in Figure 1b, builds upon the multiplication of the initial segmentation and ground truth, incorporating four encoder blocks with an AAC module, where each block applies successive convolutions with SE. The encoder block implementation includes conditional max pooling. The first block processes input directly without downsampling, while subsequent blocks apply 2 × 2 max pooling for spatial dimension reduction. Each block concludes with an SE layer that adaptively recalibrates channel responses, improving the network’s sensitivity to informative features while suppressing less relevant ones. This modification allows for more nuanced feature extraction at the deepest level of the network. Similar to Phase 1, Phase 2 also employs an ASPP module and a series of four decoder blocks, though these differ significantly in their fusion strategy. Each second decoder block implements a triple-input concatenation approach, combining upsampled features with skip connections from both the Phase 2 encoder and corresponding Phase 1 decoder outputs. This dual-phase skip connection strategy leverages the complementary strengths of both encoding paths; Phase 1’s VGG-19 features provide robust low-level cellular boundary information, while Phase 2’s encoder captures refined high-level semantic context.

For WBC segmentation, this cross-phase feature integration is crucial as it enables simultaneous preservation of fine cellular membrane details and broader morphological patterns, addressing the challenge of accurately delineating complex WBC boundaries while maintaining cellular structure integrity. The outputs from both phases are concatenated to produce the final segmentation result. This dual-phase approach, enriched with AAC and gating mechanisms, aims to capture both fine-grained cellular details and broader contextual information, potentially leading to superior segmentation performance in complex scenarios.

## 4. Experimental Results and Analysis

### 4.1. Datasets

In this study, we evaluated our method using four publicly available labeled segmentation datasets, augmented by a fifth dataset derived from the original SegPC-2021 to enhance cell-level analysis.

The original datasets included SegPC-2021 [40], CellaVision [41], JTSC [41], and Raabin-WBC [42]. The four datasets presented the challenge of classifying white blood cells into three classes (nucleus, cytoplasm, and the background) at the pixel level. Originally, SegPC-2021, comprising a total of 775 high-resolution images 2560 × 1920 and 2040 × 1536 in size, was distributed across three parts of the challenge. Specifically, 298 images were made accessible during the training phase, with 200 during the validation phase and the remaining 277 during the test phase. The reference annotations for the test set of 277 images have not been made publicly accessible. In accordance with the evaluation protocol outlined in [38], we extracted instances of white blood cells (WBCs) and created images 224 × 224 pixels in size to test the model on individual cells from the SegPC-2021 dataset. As a result, a second dataset was created, which we will refer to as the cropped SegPC-2021 dataset in our study. The CellaVision dataset contains 100 images with a resolution of 300 × 300 pixels. This dataset features ground-truth segmentation of the nucleus and cytoplasm, which was performed by a hematology expert. The JTSC dataset comprises 300 images of individual white blood cells, each with a resolution of 120 × 120 pixels. It also features ground-truth segmentation performed by an expert. This dataset was acquired by the Jiangxi Tecom Science Corporation in China. The Raabin-WBC dataset is a publicly accessible collection comprising approximately 40,000 images of normal peripheral WBCs at a size of 575 × 575 pixels, along with artifacts such as color spots. Each image has been meticulously labeled, with a significant subset annotated by two experts to ensure data accuracy and reliability. For the segmentation task, ground truth masks for nuclei and cytoplasm are provided for 1145 selected cells. The protocol followed for our work was also used by Guo et al. [31]. The split of each dataset is shown in Table A2.

**Figure 2:**
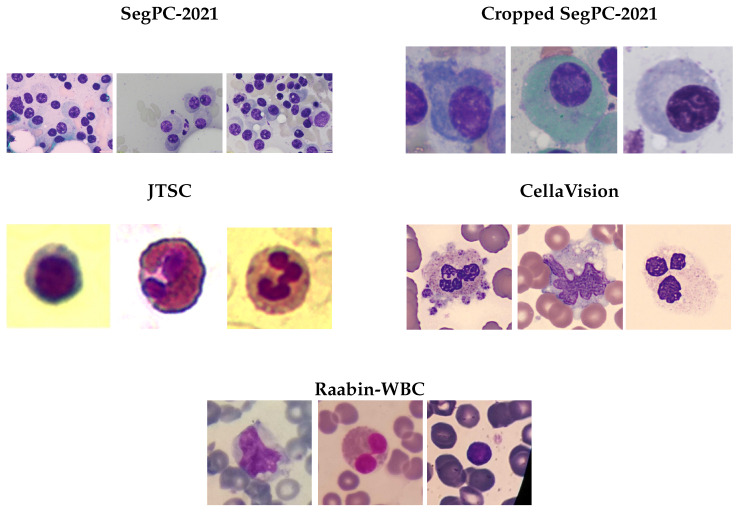
Sample of original images from different WBC datasets for model training and evaluation.

To provide a more comprehensive understanding of the datasets used in this study, we present a random selection of sample images for each dataset. These images showcase the variability in cell shape, size, and the variability in image contrast and brightness, which are crucial for training and evaluating the performance of our modified deep learning architecture. The following samples in Figure 2 illustrate the diversity and complexity of the images contained in the datasets and offer a detailed visual comparison of samples from five WBC datasets utilized for model training and assessment in medical image analysis and cell segmentation:

**(1) SegPC-2021 dataset:** This dataset provides a high concentration of purple-stained nucleated cells, presumably leukocytes, placed against a pale pink backdrop. The cells are clearly delineated and distinct, exhibiting strong contrast. The staining is uniform, rendering cell borders distinctly observable. The image quality is excellent, featuring a strong focus, minimum background noise, and thus enhanced accurate segmentation.

**(2) Cropped SegPC-2021 dataset:** This dataset was derived from the original SegPC-2021 dataset. Consequently, the evaluation of the model was applied to the WBC instance level without additional processing of the images.

**(3) The JTSC dataset:** This dataset consists of photographs that emphasize individual cells at a high magnification. The cells display distinct internal architectures, comprising nuclei and cytoplasmic features. The background ranges from pale yellow to white, offering superior contrast. This dataset seems suitable for comprehensive cellular component analysis, although it may not accurately reflect the intricacies of cell distribution in a standard blood smear.

**(4) CellaVision dataset:** This collection displays grouped leukocytes exhibiting complex nuclear patterns. The cells exhibit greater sizes and details compared with the other datasets, including discernible lobes and granules. The background includes red blood cells, complicating the segmentation operation. This dataset presumably comprises photos from automated digital morphology systems, providing high-resolution representations of certain cell clusters. These datasets exhibit various complications.

**(5) Raabin-WBC dataset:** [42] To introduce diversity, the dataset includes images captured from various smears using two different cameras and microscopes. This variability presents a challenge for deep learning models, as they must generalize across differing imaging conditions and equipment. The Raabin-WBC dataset supports multiple machine learning tasks, including classification, detection, segmentation, and localization of WBCs.

The variation in image attributes—such as brightness, contrast, cell distribution, and smear preparation—across these datasets is crucial for developing robust WBC segmentation algorithms. It ensures that models trained on these data can adapt to real-world clinical samples, accounting for differences in staining techniques, microscopy settings, and sample preparation methods across different laboratories. As a result, these datasets present several challenges:

- Cell density: Ranges from single cells (JTSC) to dense aggregates (SegPC-2021 and CellaVision).
- Staining variation: Uniform in SegPC-2021 and more inconsistent in Raabin-WBC.
- Background: Clean in JTSC and SegPC-2021 and more intricate with RBCs in CellaVision.
- Magnification: Varies from low (SegPC-2021) to high (JTSC and CellaVision).

### 4.2. Evaluation Metric

#### 4.2.1. Intersection over Union (IoU)

The intersection over union (IoU) or Jaccard index measures the overlap between the predicted (*A*) and ground truth (*B*) segmentations:

(4) $$\mathrm{IoU}=\frac{\left|A\cap B\right|}{\left|A\cup B\right|}$$

where the following definitions apply:

- A∩B is the intersection of *A* and *B*.
- A∪B is the union of *A* and *B*.

The IoU ranges from 0 (no overlap) to 1 (perfect overlap).

#### 4.2.2. Dice Similarity Coefficient (DSC)

The DSC measures the overlap, emphasizing the intersection size:

(5) $$\mathrm{DSC}=\frac{2|A\cap B|}{\left|A|+|B\right|}=\frac{2TP}{2TP+FP+FN}$$

where the following definitions apply:

- TP represents true positives.
- FP represents false positives.
- FN represents false negatives.

The DSC ranges from 0 to 1, with 1 indicating perfect agreement.

#### 4.2.3. Accuracy

Accuracy measures the proportion of correctly classified pixels:

(6) $$\mathrm{Accuracy}=\frac{TP+TN}{TP+TN+FP+FN}$$

where the following definition applies:

- TN represents true negatives.

The accuracy ranges from 0 to 1, with 1 indicating all predictions were correct.

#### 4.2.4. Surface Distance Metrics

To quantitatively evaluate boundary fidelity, we employed two established surface distance metrics: the 95th percentile Hausdorff distance (HD95) and the average symmetric surface distance (ASSD). These are defined as follows:

(7) $$HD95\left(A,B\right)=\max\left(K_{95\%}^{a\in A}\left\{\min_{b\in B}∥a-b∥\right\},K_{95\%}^{b\in B}\left\{\min_{a\in A}∥b-a∥\right\}\right)$$

(8) $$ASSD\left(A,B\right)=\frac{1}{\left|A|+|B\right|}\left(\sum_{a\in A}\min_{b\in B}∥a-b∥+\sum_{b\in B}\min_{a\in A}∥b-a∥\right)$$

where the following definitions apply:

- *A* is the set of surface points of the ground truth segmentation.
- *B* is the set of surface points of the predicted segmentation.
- *a* is an individual point belonging to set *A* (a∈A).
- *b* is an individual point belonging to set *B* (b∈B).
- $K_{95\%}$ is the operator that calculates the 95th percentile from a set of distances.

### 4.3. Implementation Details

All experiments were implemented using the PyTorch 2.4.0 framework on a single NVIDIA RTX 3060 (12 GB) GPU, an Intel Core i7-10th Gen 8-core CPU, and 64 GB of RAM. The Dice loss function was used, and all models were trained with the Adam optimizer [43] at a learning rate of 1 × 10^−4^. To adjust the learning rate, we applied polynomial learning rate decay as a scheduler.

Batch sizes were set to 4 or 16, depending on the image size. For the SegPC-2021 dataset (which contains multiple cells per image), the input images were resized to 512 × 512. In contrast, the image size for the other datasets was fixed at 224 × 224.

All experiments were conducted on the same train, validation, and test sets to ensure fair comparisons. Additionally, state-of-the-art comparisons from previous papers were included in the tables as they were reported.

### 4.4. Data Augmentation

Due to the lack of datasets for WBC segmentation tasks, we incorporated data augmentation methods to reduce the risk of model overfitting. Our augmentation method included geometric transformations (horizontal and vertical flipping and rotation), each with a probability of 0.5. We also included color space augmentations (Gaussian noise and brightness alteration) with the same probability. The augmentation techniques were carefully adjusted to introduce a set of variations without compromising data integrity, thereby enhancing the model’s robustness. With these techniques, we aimed to enhance the network’s ability to generalize well so that the features learned would be insensitive to typical distortions in real-world environments.

### 4.5. Results

The evaluation, as presented in Table 2, Table 3, Table 4 and Table 5 demonstrates the superior performance of our proposed GAAD-U-Net architecture compared with DCSAU-Net and GA2Net across five diverse WBC segmentation datasets. GAAD-U-Net achieved the lowest HD95 and ASSD values in 7 out of 10 metric-dataset combinations, indicating its enhanced capability for precise boundary delineation. Notably, GAAD-U-Net exhibited substantial improvements on the SegPC-2021 dataset, with HD95 and ASSD reductions of approximately 11.7% and 9.9%, respectively, compared with DCSAU-Net, underscoring its robustness in handling complex cellular arrangements. The model also showed remarkable performance on CellaVision, achieving HD95 and ASSD values of 1.1974 and 0.1912, representing improvements of 35.7% and 50.7%, respectively, over DCSAU-Net. While DCSAU-Net performed best on the Raabin-WBC dataset, with an HD95 of 0.3380 and ASSD of 0.0681, and GA2Net achieved the lowest ASSD on JTSC (0.0663), GAAD-U-Net maintained competitive or superior performance across all other evaluations. These results validate the efficacy of GAAD-U-Net’s dual-phase AAC integration and gating mechanisms in producing more accurate boundary representations, which is critical for reliable WBC morphological analysis in clinical applications.

**Figure 3:**
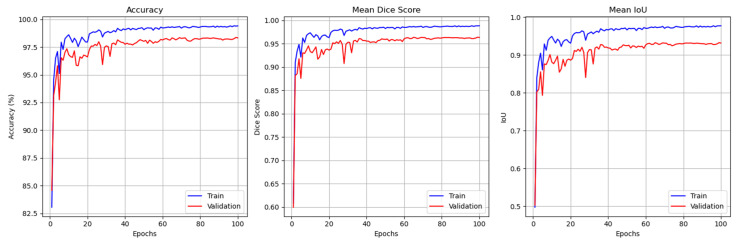
CellaVision performance monitoring plot for training and validation (from left to right). Accuracy, mean DSC, and mean IoU.

Figure 3 illustrates the evolution of these metrics over the 100 training epochs for both the training and validation datasets. The left plot shows that the training and validation accuracy consistently improved, converging to 99% and 97.5%, respectively. These results highlight GAAD-U-Net’s ability to accurately segment cellular boundaries, even in challenging cases. The DSC values, shown in the center plot, stabilized at approximately 0.98 and 0.95 for the training and validation datasets, respectively. This indicates that the model maintained a high spatial overlap between the predicted masks and the ground truth annotations. Minor fluctuations in the validation DSC reflect tissue variability and potential challenges associated with generalization to unseen samples. The IoU scores shown in the right plot averaged about 0.97 for training and about 0.93 for validation. These high scores confirm the accuracy of our segmentation.

**Figure 4:**
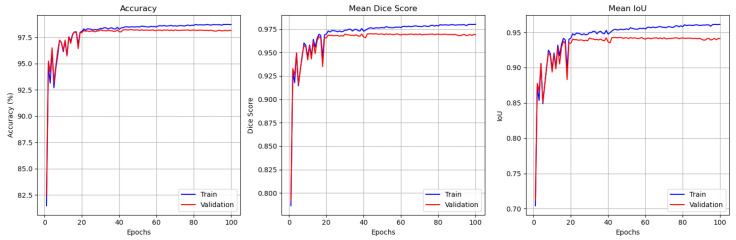
JTSC performance monitoring plot for training and validation (from left to right). Accuracy, mean DSC, and mean IoU.

For the JTSC dataset, the results are presented in Figure 4, which illustrates the training and validation performance of the GAAD-U-Net model over 100 training epochs. The first plot illustrates the accuracy, showing a steady improvement in training accuracy that stabilized at around 99%, while validation accuracy leveled off at approximately 98%. The second plot illustrates the mean DSC, which is indicative of the model’s segmentation quality, showing that the training DSC stabilized above 0.98, while the validation DSC was about 0.96. The third plot displays the mean intersection over union (IoU), which followed a similar trend, with the training IoU exceeding 0.96 and the validation IoU approaching 0.94. The slight discrepancy between the training and validation metrics suggests minimal overfitting; however, the results underscore the model’s robust and consistent performance in segmentation tasks for the JTSC dataset.

**Figure 5:**
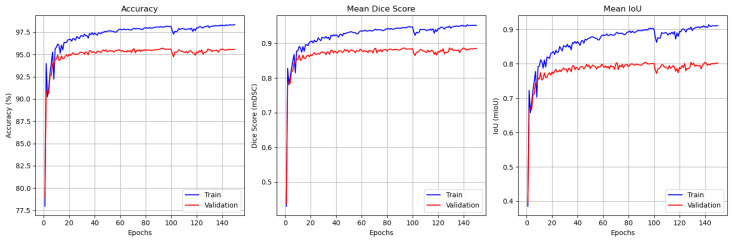
SegPC-2021 performance monitoring plot for training and validation (from left to right). Accuracy, mean DSC, and mean IoU.

The GAAD-U-Net training progression on the SegPC-2021 dataset, spanning over 150 epochs, as shown in Figure 5, demonstrated consistent improvement across all evaluated metrics, with both the training and validation curves exhibiting clear convergence patterns. The accuracy metric revealed strong performance, with the training accuracy stabilizing at nearly 99% and the validation accuracy maintained at approximately 95% after initial oscillations, indicating effective generalization with minimal overfitting. The mean DSC followed a similar trajectory, with training and validation scores converging to approximately 0.92 and 0.85, respectively. The mean IoU metric further validates the model’s robust performance, showing training IoU stabilization at 0.89 and validation IoU stabilization at 0.80, collectively demonstrating the model’s capability to effectively segment cervical cells while maintaining good generalization properties across unseen data.

**Figure 6:**
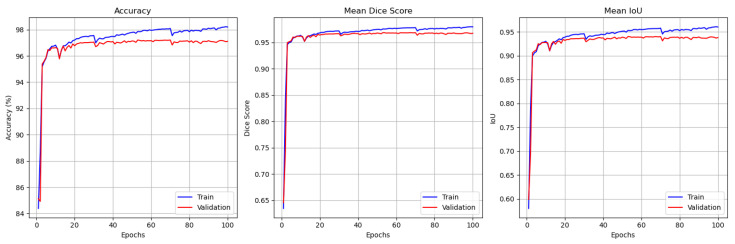
Cropped SegPC-2021 performance monitoring plot for training and validation (from left to right). Accuracy, mean DSC, and mean IoU.

The training progression for the cropped version of the SegPC-2021 dataset is illustrated in Figure 6. The plots show rapid and stable convergence, with both training and validation accuracy reaching above 96%. The mean DSC and IoU scores for the validation set stabilize at approximately 0.95 and 0.93, respectively, demonstrating the model’s generalization ability when segmenting individual, cropped WBC.

**Figure 7:**
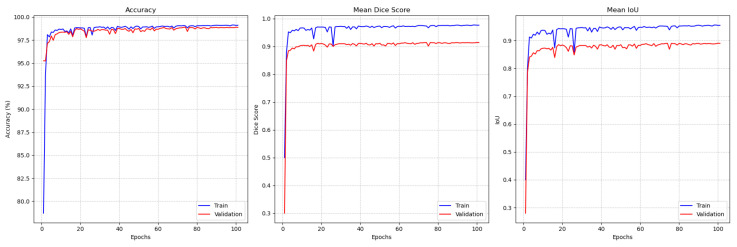
Raabin-WBC performance monitoring plot for training and validation (from left to right). Accuracy, mean DSC, and mean IoU.

Finally, the performance on the Raabin-WBC dataset is depicted in Figure 7. The model demonstrates robust learning, with the training and validation curves for accuracy, DSC, and IoU tracking each other closely. This indicates a lack of significant overfitting. The validation accuracy converges around 98.8%, with a validation DSC of approximately 0.91 and IoU around 0.89, confirming the model’s effectiveness across this dataset as well.

### 4.6. Prediction Visualizations

**Figure 8:**
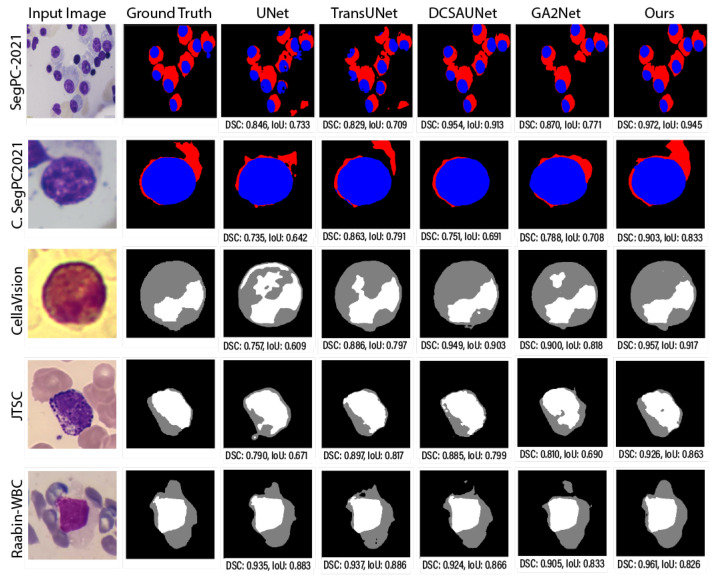
Visual comparison of WBC segmentation results across multiple datasets (from top to bottom): SegPC-2021 (whole), SegPC-2021 (cropped), JTSC, and CellaVision. Nuclei and cytoplasm are shown in blue and red, respectively, for both SegPC-2021 variants (rows 1 and 2). For CellaVision, JTSC, and Raabin-WBC, nuclei and cytoplasm are displayed in white and gray, respectively.

In comparing the segmentation outputs in Figure 8, several notable observations emerged regarding accuracy, boundary delineation, and morphological fidelity. First, although DCSAU-Net [36] recovered the general shape and location of nuclei (blue and white) and cytoplasm (red and gray), it exhibited slight over- and under-segmentation in certain regions. For instance, the bounding boxes highlight areas where cytoplasmic boundaries either encroached upon adjacent cells (over-segmentation) or failed to capture the complete cell contour (under-segmentation), potentially leading to fragmented or smeared shapes. Such errors were particularly visible when the cells were tightly clustered or when subtle intensity variations challenged the model’s capability to distinguish cell boundaries accurately.

In contrast, the proposed GAAD-U-Net model showed more consistent segmentation performance, as evidenced by closer alignment with the ground truth outlines and fewer artifacts within the highlighted boxes. The refined delineation around the nuclei and cytoplasmic regions suggests that GAAD-U-Net’s architecture better preserves spatial context and fine details. Likely, its attention mechanisms (or equivalent advanced modules) facilitate learning of more discriminative features, improving its ability to capture subtle intensity transitions at the cell periphery. This enhanced boundary adherence is crucial in cellular morphological analyses, where small segmentation discrepancies can propagate into significant downstream errors, such as altering the cell size or shape statistics.

### 4.7. Ablation Study

To evaluate the effectiveness of our proposed GAAD-U-Net architecture, we conducted an ablation study to analyze the individual contributions of its two key components: the AAC strategy and the gating block. We compared four configurations across five datasets using the accuracy, DSC, and IoU metrics. This evaluation is detailed in Table 6.

#### 4.7.1. AAC Integration Impact

The integration of the AAC strategy into our baseline model demonstrated consistent performance improvements across all datasets. When implemented as a standalone enhancement (AAC+Base), this module effectively improved the model’s ability to capture long-range dependencies and focus on relevant features, as presented in Table 6. On the SegPC-2021 dataset, incorporating the AAC module increased the DSC by 2.3%. Similarly, the mean IoU improved by 1.94%. The AAC module had a significantly greater impact on the Cropped SegPC-2021 dataset, elevating the DSC and mean IoU by 6.7% and 14.0%, respectively. For the JTSC dataset, there was an observed boost due to AAC integration in terms of the DSC and mean IoU, representing improvements of 1.0% and 3.5%, respectively. The CellaVision dataset showed substantial gains with AAC implementation, with a 1.2% DSC increase and 1.0% mean IoU improvement. On the Raabin-WBC dataset, the AAC module integration showed minor improvements in both metrics. The AAC component effectively enhanced the model’s feature representation capabilities across diverse datasets by incorporating attention mechanisms that helped the network focus on the most relevant image regions while suppressing noise and irrelevant information. The attention mechanism’s ability to establish global contextual relationships among features proves particularly valuable in segmenting white blood cells with complex morphologies.

#### 4.7.2. Gating Module Significance

The gating module was designed to adaptively control information flow through the network, allowing it to filter irrelevant information and focus on the features most critical for accurate segmentation. As Table 6 shows, when the gating module was implemented alone with the base architecture (Gating+Base), it exhibited varying degrees of improvement across different datasets. On the SegPC-2021 dataset, the gating module marginally decreased performance compared with the baseline, with a 0.8% drop in the DSC and a 0.32% decrease in the mean IoU. However, on other datasets, the gating module demonstrated positive contributions. For the Cropped SegPC-2021 dataset, incorporating the gating module resulted in a 6.8% increase in the DSC and a substantial 14.0% improvement in the mean intersection over union (IoU). On the JTSC dataset, incorporating the gating module showed minor improvement in the DSC and a 1.3% improvement in the mean IoU. On the CellaVision dataset, our model showed minor improvement when the gating module was used, with a slight increase in the DSC and mean IoU scales. For the Raabin-WBC dataset, the integration of the gating module resulted in DSC and mean IoU values that were nearly identical to those of the baseline, indicating only minimal differences. Notably, while the gating module alone showed mixed results, its true potential was realized when combined with the AAC strategy in the full GAAD-U-Net architecture. This suggests that the gating module’s adaptive feature filtering capabilities complement the AAC’s attention mechanism. To this end, gating is able to create a synergistic effect that enhances overall performance beyond what either component can achieve independently. The gating module appeared to be particularly effective on datasets with complex backgrounds and cellular morphologies, where controlling information flow becomes crucial for accurate segmentation.

## 5. Discussion

Many deep learning segmentation methods were used in the field of medical imaging, including microscopic images and WBC images. The encoder-decoder U-Net [19] architecture is well known for this task, allowing the fusion of low-level to high-level semantic information through skip connections. Double-U-Net [22], as its name suggests, is one of its variants that utilizes two U-Nets to refine the initial segmentation through a second phase. Our approach employs attention-augmented convolution in both U-Nets to retain as much global and local salient information as possible within the image and then refine it with a gating module before feeding the output to the ASPP module, which focuses on extracting refined information from different scales. Furthermore, our work highlighted the impact of the AAC module and the gating module on the performance using the DSC and IoU metrics stated in Table 6.

The experimental results, as shown in the tables, indicate that the GAAD-U-Net model achieved good performance due to the integration of AAC, as it extends the traditional Double U-Net [22] receptive field, thereby augmenting its ability to capture global information. In addition, Table 6 shows that when the sole use of the gating module with the traditional Double U-Net architecture did not yield a significant improvement, its true potential was observed when combined with the AAC strategy in the full GAAD-U-Net architecture. This suggests that the gating module’s adaptive feature filtering capabilities complement the AAC’s attention mechanism. Based on this, gating is able to create a synergistic effect that enhances overall performance beyond what either component can achieve independently. The gating module appeared to be particularly effective on datasets with complex backgrounds and cellular morphologies, where controlling information flow becomes crucial for accurate segmentation.

**Figure 9:**
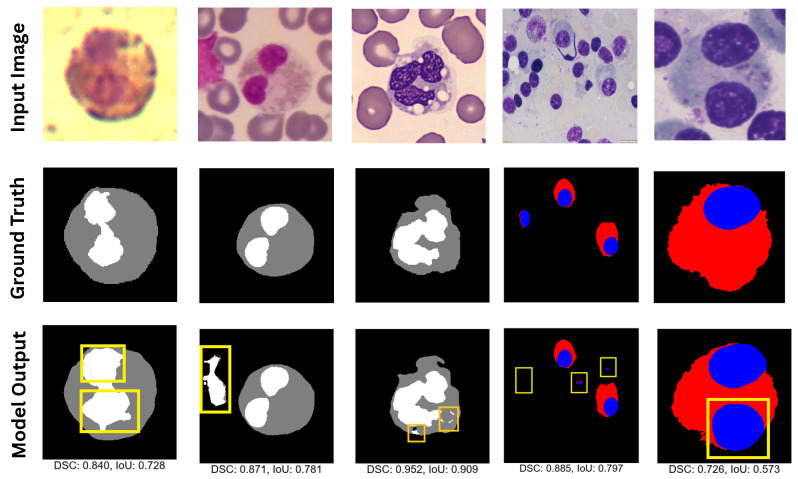
Failure cases where GAAD-U-Net struggled to segment the target WBC in images across five different WBC image segmentation datasets. Failure regions are bounded inside yellow boxes. Nuclei and cytoplasm are shown in blue/white and red/grey.

To further illustrate the improvement of the GAAD-U-Net model in WBC image segmentation, we present a visual comparison of the results of all models for challenging images that displayed individual cells and multiple cells in a single image, as shown in Figure 8. The qualitative results demonstrate that our proposed model generated segmentation masks that more effectively captured foreground details in low-quality images, such as those with incomplete staining or obscurity, compared with other state-of-the-art methods. However, our model struggled in some complex scenarios that contained multiple targets or extremely intricate details, as shown in Figure 9. In summary, our model demonstrated superior performance in white blood cell segmentation, accurately capturing cell boundaries and achieving an average improvement of 1.4% in the DSC and 1.7% in the mIoU, Dice, and IoU scores compared with other state-of-the-art segmentation methods. Our model achieved superior accuracy and better boundary delineation than existing methods, establishing a new benchmark in the field of WBC image segmentation.

## 6. Conclusions

In this work, we introduced GAAD-UNet, a novel architecture for WBC segmentation that builds upon a double encoder-decoder (Double U-Net) framework. Our approach integrates AAC blocks with a gating module at the bottleneck of both encoder-decoder branches, enabling the network to capture rich local and global contextual features while filtering out irrelevant information. This design improves the delineation of complex structures, particularly excelling in the segmentation of the cytoplasm, a challenging task due to the subtle intensity variations and a smear-like appearance in microscopic images.

Extensive evaluations across five diverse datasets demonstrated that GAAD-U-Net achieved superior performance compared with current state-of-the-art methods, as evidenced by significant improvements in the DSC and IoU metrics. Our model robustly handled individual cropped WBCs and images containing multiple cells, underscoring its versatility and potential for broad clinical application.

However, these performance gains come at the cost of a higher parameter count and increased computational complexity, which may result in slower inference speeds and pose challenges in resource-constrained environments. Future work will focus on optimizing the architecture through model compression techniques and adaptive attention strategies, aiming to reduce computational overhead while preserving or even enhancing segmentation accuracy. This detailed exploration not only highlights the strengths of GAAD-U-Net but also outlines clear avenues for further refinement, paving the way for its practical deployment in clinical settings.

## Figures and Tables

**Table 1:** Key differences between our model and baseline, AA-U-Net, and DCSAU-Net.

Feature	Double U-Net	AA-U-Net	DCSAU-Net	GAAD-U-Net (Ours)
**Base**	Double U-Net	U-Net	U-Net	Double U-Net
**Attention**	None	AAC	CSA	AAC
**Mechanism**	N/A	Multi-Head Self-Attention	Multi-Path Soft Attention	Multi-Head Self-Attention
**Placement**	N/A	Bottleneck	Encoder or Decoder skips	Both bottlenecks with Gating, ASPP
**Attention Enhancement Modules**	N/A	None	PFC	Gating, ASPP, Dual-Skip
**Benefit**	Dual-Phase Refinement	Wider Context	Multi-Scale Efficiency	Feature Refinement and Wider Context

**Table 2:** Comparison of segmentation methods on SegPC-2021 dataset based on accuracy, precision, recall, DSC, and mIoU. Best scores are in **bold**.

Method	Accuracy	Precision	Recall	DSC	Mean IoU
U-Net (2015) [19]	0.939	0.842	0.879	0.855	0.766
Attention U-Net (2018) [44]	0.940	0.845	0.866	0.849	0.757
R2U-Net (2018 [45]	0.933	0.852	0.831	0.834	0.744
ResU-Net++ (2019) [46]	0.934	0.838	0.858	0.840	0.736
U-Net++ (2020) [34]	0.942	0.855	0.876	0.857	0.770
Double U-Net (2020) [22]	0.937	0.833	0.896	0.858	0.763
UNet3+ (2020) [47]	0.939	0.848	0.866	0.852	0.766
TransUNet (2021) [48]	0.939	0.822	0.869	0.838	0.741
LeViT-UNet (2021) [49]	0.939	0.850	0.837	0.837	0.738
DCSAU-Net (2023) [36]	0.950	0.871	0.910	0.886	0.806
GA2Net (2024) [38]	0.953	0.866	0.793	0.877	0.793
**GAAD-U-Net (ours)**	**0.960**	**0.873**	**0.924**	**0.901**	**0.830**

**Table 3:** Comparison of model performance based on accuracy, DSC, and mean IoU on cropped SegPC-2021. Best scores are in **bold**.

Model	Accuracy	DSC	Mean IoU
U-Net (2015) [19]	0.9390	0.8808	0.8820
U-Net++ (2020) [34]	0.9420	0.9102	0.9092
Double U-Net (2020) [22]	0.9470	0.8941	0.7991
MultiResUNet (2021) [50]	-	0.8649	0.8676
TransU-Net (2021) [48]	0.9390	0.8233	0.8338
MissFormer (2023) [51]	-	0.8082	0.8209
UCTransNet (2021) [52]	-	0.9174	0.9159
DCSAU-Net (2023) [36]	0.9504	0.8860	0.8060
GA2-Net (2024) [38]	-	0.9274	0.9254
**GAAD-U-Net (Ours)**	**0.9852**	**0.9614**	**0.9330**

**Table 4:** Quantitative performance comparison of segmentation models across three public WBC datasets: CellaVision, JTSC, and Raabin-WBC. Best scores are in **bold**.

Model	CellaVision			JTSC			Raabin-WBC		
Metrics	Acc.	DSC	Mean IoU	Acc.	DSC	Mean IoU	Acc.	DSC	Mean IoU
U-Net (2015) [19]	0.9422	0.8922	0.8215	0.9720	0.9519	0.9104	0.9860	0.9068	0.8800
Double U-Net (2020) [22]	0.9792	0.9495	0.9096	0.9803	0.9621	0.9319	0.9843	0.9091	0.8832
TransUNet (2021) [48]	0.9790	0.9534	0.9145	0.9737	0.9603	0.9249	0.9870	0.9094	0.8840
DCSAU-Net (2023) [36]	0.9737	0.9460	0.9018	0.9767	0.9592	0.9235	0.9865	0.9102	0.8867
GA2Net (2024) [38]	0.9560	0.8989	0.8403	0.9812	0.9680	0.9389	0.9865	0.9055	0.8700
**GAAD-U-Net (Ours)**	**0.9830**	**0.9589**	**0.9214**	**0.9832**	**0.9714**	**0.9674**	**0.9884**	**0.9119**	**0.8885**

**Table 5:** Boundary-fidelity comparison of DCSAU-Net, GA2Net, and GAAD-U-Net across different datasets using HD95 and ASSD surface metrics. Best scores are marked in **bold**.

Model	SegPC-2021		Cropped SegPC-2021		CellaVision		JTSC		Raabin-WBC	
	HD95	ASSD	HD95	ASSD	HD95	ASSD	HD95	ASSD	HD95	ASSD
**DCSAU-Net**	11.8905	1.7358	0.5435	0.1148	1.8626	0.3877	0.6720	0.0942	**0.3380**	**0.0681**
**GA2Net**	20.2653	4.0739	0.5830	0.1092	2.1052	0.3864	0.4634	**0.0663**	0.3653	0.0693
**GAAD-U-Net**	**10.5024**	**1.5632**	**0.4453**	**0.0943**	**1.1974**	**0.1912**	**0.4211**	0.0689	0.3470	0.0706

**Table 6:** Ablation study on performance metrics across five datasets for different model variations: Base, AAC+Base, Gating+Base, and GAAD-U-Net. The best results for each dataset are highlighted in **bold**.

Dataset	Metric	Base	Base+AAC	Base+Gating	Base+AAC+Gating
SegPC-2021	Accuracy	0.9370	0.9470	0.9320	**0.9597**
	DSC	0.8580	0.8780	0.8509	**0.9011**
	Mean IoU	0.7630	0.7824	0.7598	**0.8301**
Cropped SegPC-2021	Accuracy	0.9470	0.9722	0.9722	**0.9852**
	DSC	0.8980	0.9585	0.9587	**0.9614**
	Mean IoU	0.7991	0.9112	0.9112	**0.9330**
JTSC	Accuracy	0.9285	0.9831	0.9300	**0.9832**
	DSC	0.9621	0.9713	0.9646	**0.9714**
	Mean IoU	0.9319	0.9645	0.9444	**0.9674**
CellaVision	Accuracy	0.9792	**0.9845**	0.9792	0.9830
	DSC	0.9495	**0.9611**	0.9496	0.9589
	Mean IoU	0.9096	0.9189	0.9100	**0.9214**
Raabin-WBC	Accuracy	0.9843	0.9870	0.9851	**0.9884**
	DSC	0.9091	0.9112	0.9088	**0.9119**
	Mean IoU	0.8832	0.8846	0.8822	**0.8885**

**Figure A1:**
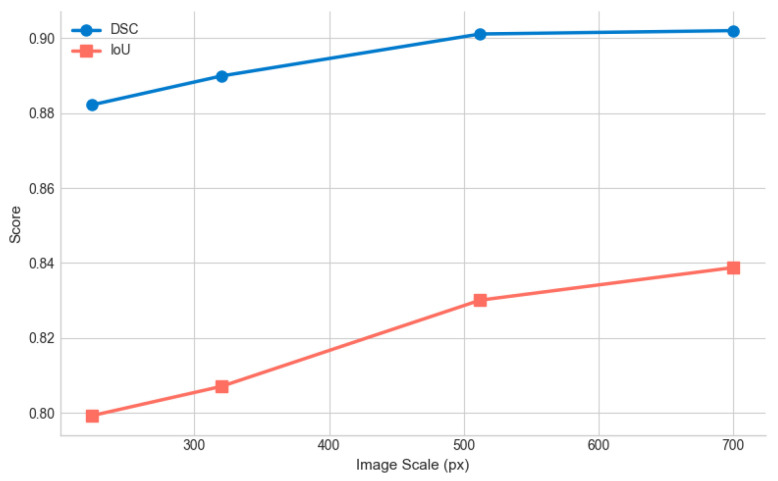
Performance sensitivity for different image scales on SegPC-2021 dataset.

**Figure A2:**
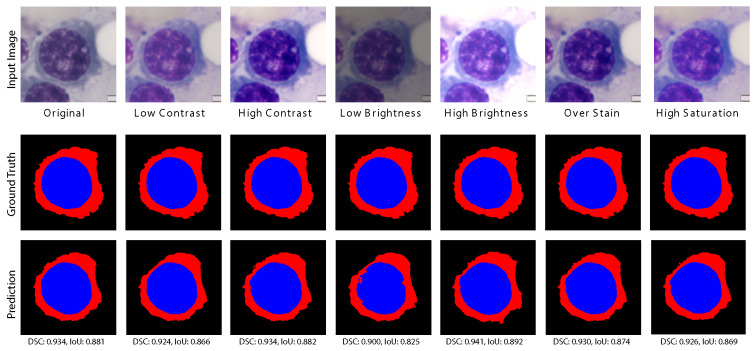
Performance of GAAD-U-Net under various color and staining conditions. DSC and IoU scores are reported for each condition test.

**Figure A3:**
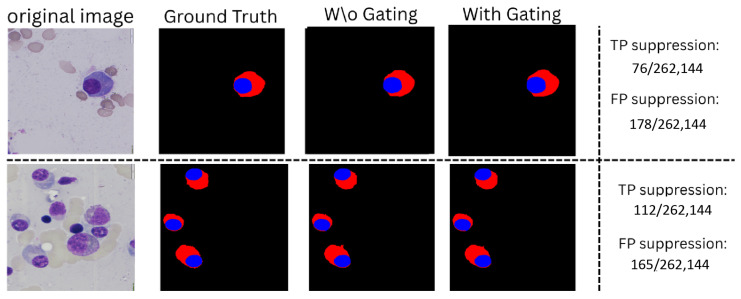
Gating mechanism pixel suppression test. Nucleus and cytoplasm masks are in blue and red, respectively.

**Figure A4:**
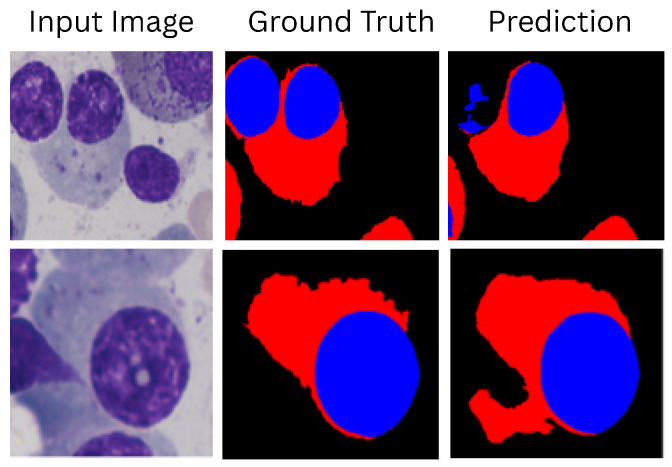
Samples where GAAD-U-Net struggled in overlapping WBC cases. Nucleus and cytoplasm masks are in blue and red, respectively.

**Figure A5:**
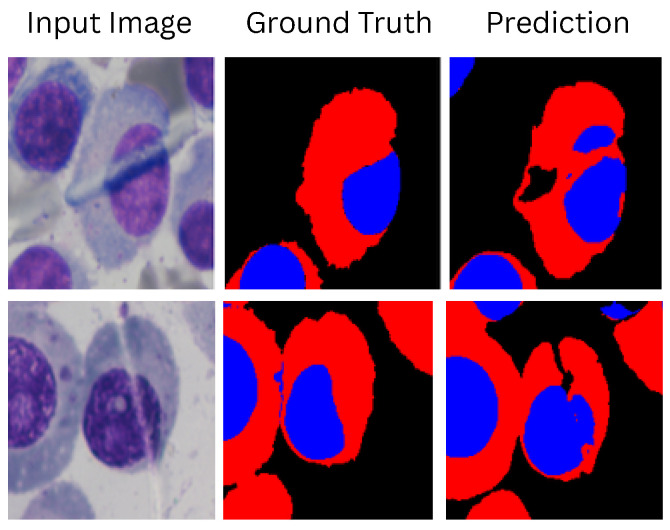
Samples where GAAD-U-Net struggled in stained WBC images. Nucleus and cytoplasm masks are in blue and red, respectively.

**Figure A6:**
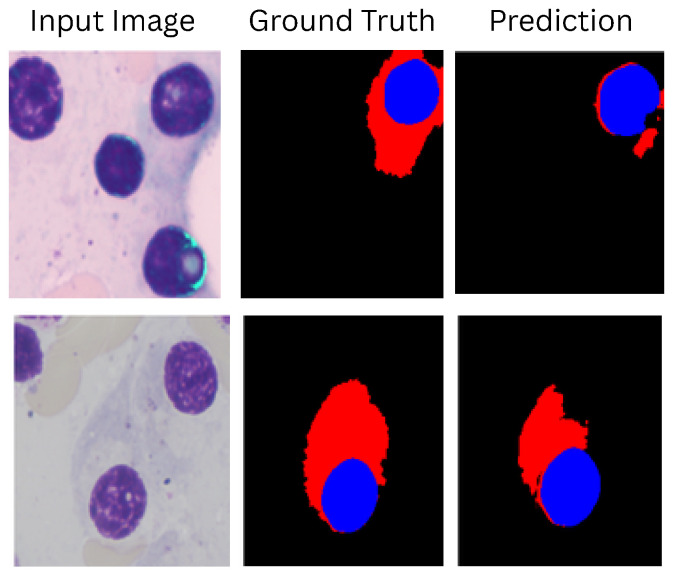
Samples where GAAD-U-Net failed to segment due to ambigious cytoplasm boundaries. Nucleus and cytoplasm masks are in blue and red, respectively.

## Data Availability

All datasets utilized in this study are publicly available and originate from previously published, ethically approved sources. No new human or animal data were collected or generated for this research. **SegPC-2021 Dataset:** Provided by the IEEE VIP Cup 2021 challenge, this dataset is publicly accessible under the challenge’s open-access terms of use, and it was employed in full compliance with its licensing conditions. The modified SegPC-2021 (cropped) dataset used in this work was derived from the publicly available version of the dataset, and no additional human subject information was introduced or modified. **Raabin-WBC Dataset:** The Raabin-WBC dataset is an open-source dataset released by Raabin Lab (Tehran, Iran) under the Creative Commons Attribution–NonCommercial 4.0 International License (CC BY-NC 4.0). All images were fully anonymized prior to release. **CellaVision and JTSC Datasets:** These datasets are publicly available, and they were used solely for non-commercial research purposes. All images were de-identified, containing no personally identifiable or sensitive patient information.

## Appendix A. Architecture

Table A1 details each stage of the proposed dual-path encoder-decoder architecture. The input tensor has a shape [4,3,224,224] (batch of four RGB images). The first encoder path is based on VGG-19 blocks, progressively increasing the channel depth from 64 up to 512 and reducing the spatial resolution accordingly. Each subsequent AAC, gating, and ASPP module further processes the feature maps at [4,512,28,28]. (ASPP expands channels to 1024) Decoder1 then upsamples the output back to the original resolution, producing an intermediate result. A matrix multiplication layer merges the encoder and the previous output, feeding into the second encoder-decoder path (blocks marked with “*” reuse weights). Finally, Decoder2 reconstructs the full-resolution prediction. The “Parameters” column lists the trainable weights in each block, illustrating the model’s complexity at each stage.

**Table A1:** Proposed architecture details with output tensor sizes and trainable block parameters.

Block	Tensor Size	Parameters
Input	[4, 3, 224, 224]	-
VGG19_block1	[4, 64, 224, 224]	38,720
VGG19_block2	[4, 128, 112, 112]	221,440
VGG19_block3	[4, 256, 56, 56]	2,065,408
VGG19_block4	[4, 512, 28, 28]	8,259,584
AAC	[4, 512, 28, 28]	2,656,720
Gating Module	[4, 512, 28, 28]	263,424
ASPP	[4, 1024, 28, 28]	20,453,376
Decoder1_block1	[4, 512, 28, 28]	9,177,600
Decoder1_block2	[4, 256, 56, 56]	2,295,040
Decoder1_block3	[4, 128, 112, 112]	574,080
Decoder1_block4	[4, 64, 224, 224]	143,680
Output_1	[4, 3, 224, 224]	195
matmul	[4, 3, 224, 224]	-
Encoder2_block1	[4, 64, 224, 224]	39,360
Encoder2_block2	[4, 128, 112, 112]	223,744
Encoder2_block3	[4, 256, 56, 56]	893,952
Encoder2_block4	[4, 512, 28, 28]	3,573,760
AAC *	[4, 512, 28, 28]	2,656,720
Gating Module *	[4, 512, 28, 28]	263,424
ASPP *	[4, 1024, 28, 28]	20,453,376
Decoder2_block1	[4, 512, 28, 28]	11,569,664
Decoder2_block2	[4, 256, 56, 56]	2,893,056
Decoder2_block3	[4, 128, 112, 112]	723,584
Decoder2_block4	[4, 64, 224, 224]	181,056
Final_output	[4, 3, 224, 224]	195

* Reused block.

## Appendix B. Dataset

Table A2 outlines the data splits for the WBC segmentation datasets used in this study. Each dataset was divided into training, validation, and test subsets, with the total number of images also reported. For some datasets, such as CellaVision and JTSC, only training and test splits were provided, as no dedicated validation set was used. Notably, Cropped SepPC-2021 represents an augmented version of SepPC-2021 with significantly more samples. These diverse datasets support a comprehensive evaluation of model performance across different WBC imaging conditions and annotation styles.

**Table A2:** Study WBC segmentation datasets protocol.

Dataset	Train	Val	Test	Total	Input Size	Augmentations	Links
SegPC-2021	360	89	49	493	512 × 512	Horizontal flip Rotation Cutout probability = 0.25	https://www.kaggle.com/datasets/sbilab/segpc2021dataset (accessed on: 20 April 2025)
Cropped SegPC-2021	1843	263	527	2633	224 × 224	Horizontal flip Vertical flip Scaling probability = 0.5	https://www.kaggle.com/datasets/sbilab/segpc2021dataset (accessed on: 20 April 2025)
CellaVision	75	–	25	100	224 × 224	Vertical flip Horizontal flip Rotation Gaussian noise Brightness alteration probability = 0.5	https://github.com/zxaoyou/segmentation_WBC (accessed on: 20 April 2025)
JTSC	225	–	75	300	224 × 224	Vertical flip Horizontal flip Rotation Gaussian noise Brightness alteration probability = 0.5	https://github.com/zxaoyou/segmentation_WBC (accessed on: 20 April 2025)
Raabin-WBC	800	112	233	1145	224 × 224	Vertical flip Horizontal flip Rotation Gaussian noise Brightness alteration probability = 0.5	https://raabindata.com/ (accessed on: 20 April 2025)

## Appendix C. Hyperparameter

Table A3b summarizes the hyperparameters used to optimize the model in this study. These parameters were determined based on recent studies on this topic.

**Table A3:** Configuration parameters for the model.

(**a**) AAC Module Parameters.	
**Parameter**	**Value**
In Channels	512
Out Channels	512
Kernel Size	3
Attention Key Dim.	32
Value Key Dim.	32
Number of Heads	4
Relative	True
Stride	1
Shape	28
(**b**) Hyperparameter Settings.	
**Hyperparameter**	**Value**
Epochs	100 (150 for segPC-2021)
Batch Size	4
Optimizer	Adam Optimizer
Initial Learning Rate	1 × 10^−4^
Weight Decay	1 × 10^−4^
Momentum	0.9
Gamma	0.5

Table A3a summarizes the configuration parameters used in the AAC module. The module operates with 512 input and output channels, employing a convolution kernel with a size of three. It incorporates an attention mechanism with four heads, where both the attention key and value dimensions are set to 32. Relative positional encoding is enabled, and the convolution stride is set to one. The feature map shape processed by the module is 28 × 28.

## Appendix D. Computational Profile

Table A4 presents a concise computational profile of the GAAD-U-Net architecture, evaluated on an RTX 3060 GPU using the THOP profiler. It details the kernel sizes, channel widths, parameter counts, per-image GFLOPs, peak VRAM usage, and latency for each encoder and decoder stage across both phases. With a total latency of 189.57 ms, 89,621,024 parameters, and a peak VRAM of 1801.8 MB, the model’s good performance is accompanied by a significant latency, reflecting the trade-off inherent in its complex, multi-stage design for enhanced feature refinement.

**Table A4:** Computational profile of GAAD-U-Net on RTX 3060.

Stage	Kernel Sizes	Channels	Params	GFLOPs	VRAM (MB)	Latency (ms)
Phase1 Encoder1 VGG1	3 × 3	3 → 64	38,720	10.12	1417.7	6.54
Phase1 Encoder1 VGG2	3 × 3	64 → 128	221,440	14.50	1385.9	6.40
Phase1 Encoder1 VGG3	3 × 3	128 → 256	2,065,408	33.82	1339.6	12.41
Phase1 Encoder1 VGG4	3 × 3	256 → 512	8,259,584	33.82	1323.4	12.56
Phase1 AAC1	3 × 3	512 → 512	2,656,848	2.72	1358.0	2.62
Phase1 Gating1	1 × 1	512 → 512	263,424	0.27	1293.3	0.48
Phase1 ASPP1	1 × 1, 3 × 3, 3 × 3, 1 × 1	512 → 1024	20,453,376	20.41	1334.4	7.93
Phase1 Decoder1 Block1	2 × 2 (up), 3 × 3, 1 × 1	1024 → 512	9,177,600	15.84	1321.4	4.14
Phase1 Decoder1 Block2	2 × 2 (up), 3 × 3, 1 × 1	512 → 256	2,295,040	31.17	1389.9	11.00
Phase1 Decoder1 Block3	2 × 2 (up), 3 × 3, 1 × 1	256 → 128	574,080	37.65	1578.5	13.91
Phase1 Decoder1 Block4	2 × 2 (up), 3 × 3, 1 × 1	128 → 64	143,680	37.72	1673.6	19.62
Phase1 output	1 × 1	64 → 3	195	0.00	50	0.01
Phase2 Encoder2 Block1	3 × 3	3 → 64	39,360	10.27	1417.7	8.03
Phase2 Encoder2 Block2	3 × 3	64 → 128	223,744	14.57	1401.9	5.64
Phase2 Encoder2 Block3	3 × 3	128 → 256	893,952	14.53	1348.6	5.33
Phase2 Encoder2 Block4	3 × 3	256 → 512	3,573,760	14.51	1326.4	3.12
Phase2 AAC2	3 × 3	512 → 512	2,656,848	2.72	1358.0	2.81
Phase2 Gating2	1 × 1	512 → 512	263,424	0.27	1293.3	0.27
Phase2 ASPP2	1 × 1, 3 × 3, 3 × 3, 1 × 1	512 → 1024	20,453,376	20.41	1334.4	8.20
Phase2 Decoder2 Block1	2 × 2 (up), 3 × 3, 1 × 1	1024 → 512	11,569,664	18.26	1334.4	5.31
Phase2 Decoder2 Block2	2 × 2 (up), 3 × 3, 1 × 1	512 → 256	2,893,056	40.84	1440.1	13.79
Phase2 Decoder2 Block3	2 × 2 (up), 3 × 3, 1 × 1	256 → 128	723,584	47.32	1707.3	18.02
Phase2 Decoder2 Block4	2 × 2 (up), 3 × 3, 1 × 1	128 → 64	181,056	47.40	1801.8	21.44
Final output	1 × 1	64 → 3	195	0.00	50	0.01
Total/Peak	-	-	89,621,024	469.14	1801.8	189.57

In our comparative evaluation of the resolution pipeline presented in Table A5, we observed a pronounced throughput escalation from 9.15 to 28.98 images per second and a commensurate latency reduction from 109.34 to 34.51 ms per image when downsampling from 512 × 512 to 224 × 224, underscoring the quadratic sensitivity of convolutional architectures to spatial dimensionality. Augmentation emerged as the dominant bottleneck at higher resolutions (550.73 ms vs. 103.86 ms per sample), attributable to pixel-intensive transformations, while inference scaled subquadratically (190.83 ms vs. 66.48 ms) due to memory-bound feature propagation; both eclipsed negligible overheads like batch loading. For real-world application, it is necessary to navigate a quintessential accuracy-efficiency dialectic, privileging 512 × 512 for granular semantic fidelity in offline regimes yet favoring 224 × 224 for real-time viability, potentially augmented by multi-scale ensembles to temper representational losses.

**Table A5:** Pipeline step timing comparison between 224 × 224 and 512 × 512 image sizes.

Pipeline	Time (ms) 512 × 512	Time (ms) 224 × 224
Augmentation time per sample	550.73	103.86
Initialization and checkpoint loading	1400.13	1399.34
GPU batch loading time	1.85 (batch of 4)	1.42 (batch of 16)
Inference	190.83	66.48
Throughput	9.15 images/second	28.98 images/second
Average latency	179.34 ms per image	64.51 ms per image

## Appendix E. Extended Ablation Study

The ablation study shown in Table A6 examined head depth sweeps of 1, 2, 4, 8, and 16 with mean ±95% confidence intervals of the DSC and IoU computed across all five datasets. The results reveal that increasing the number of attention heads in the GAAD-U-Net architecture did not necessarily enhance the model’s segmentation performance for WBCs. Employing 16 heads, which processed the largest feature map expansions, exhibited a decline in performance compared with configurations with fewer heads in some cases, such as a sharp drop to a 0.6444 DSC in Cropped SegPC-2021 and a reduction to a 0.9000 DSC in the CellaVision dataset. This aligns with established insights that excessive heads in multi-head attention mechanisms can introduce redundancy and overfitting. In contrast, projections with moderate head counts preserved the granular boundary details critical for accurate WBC delineation. While dataset-specific analyses indicated that Raabin-WBC benefited more from eight heads (0.9169 DSC), the four-head configuration yielded superior mean IoU and Dice scores in datasets like SegPC-2021 (0.9011 DSC) and Cropped SegPC-2021 (0.9614 DSC). Overall, four-head attention outperformed the others in aggregated metrics for Cropped SegPC-2021 and SegPC-2021 and maintained moderate results across the rest dataset. while JTSC showed stable high performance across all configurations, nearing a 0.97 DSC, leading four-head attention to achieve the optimal balance, delivering the highest cross-dataset performance.

**Table A6:** Ablation study on the number of AAC heads across five WBC datasets. Metrics are reported as DSC and IoU with 95% confidence intervals.

Dataset	Metric	1 Head			2 Heads			4 Heads			8 Heads			16 Heads		
		Mean	Lower	Upper	Mean	Lower	Upper	Mean	Lower	Upper	Mean	Lower	Upper	Mean	Lower	Upper
Cropped SegPC-2021	DSC (%)	0.9607	0.9578	0.9635	0.9594	0.9574	0.9621	0.9614	0.9534	0.9694	0.8884	0.8786	0.8981	0.6444	0.6197	0.6690
	IoU (%)	0.9257	0.9205	0.9308	0.9323	0.9279	0.9366	0.9330	0.9229	0.9431	0.7333	0.7221	0.7444	0.5343	0.5170	0.5516
SegPC-2021	DSC (%)	0.8862	0.8615	0.9109	0.8890	0.8632	0.9149	0.9011	0.8761	0.9261	0.8790	0.8571	0.9109	0.8849	0.8581	0.9116
	IoU (%)	0.8044	0.7646	0.8442	0.8198	0.7686	0.8509	0.8301	0.7964	0.8638	0.8023	0.7599	0.8446	0.8035	0.7614	0.8455
CellaVision	DSC (%)	0.8914	0.8532	0.9295	0.9136	0.8936	0.8936	0.9589	0.9564	0.9614	0.9515	0.9490	0.9540	0.9251	0.9156	0.9346
	IoU (%)	0.8143	0.7566	0.8721	0.8500	0.8320	0.8680	0.9214	0.9158	0.9270	0.9282	0.9226	0.9338	0.8714	0.8594	0.8834
JTSC	DSC (%)	0.9695	0.9640	0.9749	0.9712	0.9662	0.9764	0.9714	0.9689	0.9739	0.9719	0.9668	0.9770	0.9720	0.9671	0.9770
	IoU (%)	0.9415	0.9314	0.9516	0.9449	0.9354	0.9543	0.9674	0.9618	0.9730	0.9460	0.9365	0.9556	0.9462	0.9369	0.9556
Raabin-WBC	DSC (%)	0.8643	0.8618	0.8668	0.9002	0.8972	0.9032	0.9119	0.9099	0.9139	0.9170	0.9144	0.9194	0.9152	0.9128	0.9176
	IoU (%)	0.7886	0.7830	0.7942	0.8795	0.8740	0.8850	0.8873	0.8804	0.8967	0.8988	0.8944	0.9032	0.8911	0.8865	0.8957

Table A7 presents the evaluation of AAC integration in GAAD-U-Net phases. Being configured with AAC in Phase 1, Phase 2, or both outperformed the baseline (no AAC), confirming attention enhancements for a cell’s delineation. Dual-phase AAC achieved peak performance (>0.90 DSC and IoU on most datasets), leveraging synergy for spatial refinement and semantic stability. Phase 1 excelled on variable morphology datasets like Cropped SegPC-2021 (0.9620 DSC, 0.9755 IoU) and Raabin-WBC (0.9124 DSC, 0.9008 IoU), and AAC in both phases did not yield superior results on these two datasets as the additional Phase 2 attention may have introduced redundancy or over-processing, potentially interfering with the optimal feature representations already established in Phase 1 for these specific dataset characteristics. Nonetheless, AAC demonstrated robust performance across all configurations, particularly when integrated in both phases, underscoring its efficacy for WBC segmentation tasks.

**Table A7:** Ablation study for AAC integration for Phase 1 and Phase 2 in GAAD-U-Net architecture. Entries marked with ✓ indicate AAC application in the respective phase, while ✗ marks indicate its absence. Reported metrics are mean DSC and mean IoU across five WBC datasets, where bold values highlight highest results.

Phase 1	Phase 2	SegPC-2021		Cropped SegPC-2021		CellaVision		JTSC		Raabin-WBC	
		DSC	IoU	DSC	IoU	DSC	IoU	DSC	IoU	DSC	IoU
✗	✗	0.8580	0.7630	0.8980	0.7991	0.9495	0.9096	0.9621	0.9319	0.9091	0.8832
✓	✗	0.8796	0.7966	**0.9620**	**0.9755**	0.9523	0.9129	0.9672	0.9526	**0.9124**	**0.9008**
✗	✓	0.8807	0.8054	0.9589	0.9733	0.9542	0.9155	0.9630	0.9437	0.9099	0.8844
✓	✓	**0.9011**	**0.8301**	0.9614	0.9740	**0.9589**	**0.9214**	**0.9714**	**0.9674**	0.9119	0.8885

## Appendix F. Generalization Tests

### Appendix F.1. Cross-Dataset Evaluation

For explicit cross-dataset generalization evaluation, our leave-one-out evaluation across five WBC datasets (Table A8) revealed a cross-DSC averaging 32–41% for the models and a 56–68% retention loss from the peak potential, which highlights the domain gaps in staining and scale. DCSAU-Net trailed at a 32% average cross-DSC (34% retention), with its dilated convolutional blocks and spatial attention modules faltering in CellaVision-to-whole-SegPC shifts (0.3% DSC and just 0.3% retention) as the attention failed to suppress smear-wide debris, amplifying noise in dense regions. GA2Net improved to a 38% cross-DSC (40% retention), where it achieved high scores on JTSC tests (76% DSC from Cropped SegPC training with 82% retention) due to the spatial gates suppressing multi-scale backgrounds, but it collapsed to 73% on the whole-smear transfers from cropped sources (24% DSC) due to overreliance on isolated-cell foreground priors. Our GAAD-U-Net topped out at a 41% cross-DSC (44% retention), surging 28% over the baselines on Raabin-WBC to CellaVision (78% DSC, 83% retention) via AAC integration in the phases’ bottleneck, which sharpened ambiguous boundaries, while the gating mechanism preserved 65% of the cropped gains over the whole-smear contrasts. This validates its anomaly-handling blocks for clinical shifts, priming a 10–15% uplift with scale adaptations.

**Table A8:** Cross-dataset generalization evaluation on different WBC datasets. Best scores are in **bold**, and in-domain results were not considered.

Model	Train Dataset	Test: SegPC-2021		Test: Cropped SegPC-2021		Test: CellaVision		Test: JTSC		Test: Raabin-WBC	
		DSC	IoU	DSC	IoU	DSC	IoU	DSC	IoU	DSC	IoU
**DCSAU-Net**	SegPC-2021	0.8860	0.8060	0.2689	0.2056	0.2990	0.2638	0.3050	0.2509	0.3033	0.2778
	Cropped SegPC-2021	0.1546	0.1170	0.8860	0.8060	0.3931	0.3310	0.4206	0.3250	0.4875	0.4167
	CellaVision	0.0031	0.0025	0.1889	0.1206	0.9460	0.9018	0.3390	0.2569	0.5521	0.4632
	JTSC	**0.0578**	**0.0349**	0.6722	0.5344	0.3362	0.2790	0.9592	0.9235	0.3678	0.3214
	Raabin-WBC	0.0475	0.0204	0.5330	0.4451	0.5567	0.4697	0.3563	0.2979	0.9102	0.8867
**GA2Net**	SegPC-2021	0.8770	0.7930	**0.3748**	**0.2800**	0.5712	0.5042	**0.4536**	**0.3145**	**0.4536**	**0.3875**
	Cropped SegPC-2021	0.2437	0.1838	0.9274	0.9254	**0.4383**	**0.3582**	**0.7649**	**0.6611**	0.4304	0.3754
	CellaVision	0.0207	0.0113	0.2276	0.1525	0.8989	0.8403	**0.4978**	**0.4109**	0.6583	0.5581
	JTSC	0.0373	0.0284	0.5739	0.4425	**0.3741**	**0.3275**	0.9680	0.9389	0.3849	0.3507
	Raabin-WBC	0.0499	**0.0276**	0.5172	0.4314	0.7162	0.6175	**0.4374**	**0.3837**	0.9055	0.8700
**GAAD-UNet**	SegPC-2021	0.9011	0.8301	0.3397	0.2444	**0.6059**	**0.5376**	0.2719	0.2299	0.3024	0.2769
	Cropped SegPC-2021	**0.2667**	**0.1955**	0.9614	0.9330	0.4300	0.3798	0.7381	0.6396	**0.4380**	**0.3784**
	CellaVision	**0.0376**	**0.0293**	**0.3278**	**0.2569**	0.9589	0.9214	0.4369	0.3611	**0.7839**	**0.7176**
	JTSC	0.0422	0.0318	**0.7427**	**0.6231**	0.3524	0.3043	0.9714	0.9674	**0.3937**	**0.3592**
	Raabin-WBC	**0.0513**	0.0265	**0.5408**	**0.4574**	**0.7822**	**0.6881**	0.3575	0.3060	0.9119	0.8885

### Appendix F.2. Scale and Color Sensitivity

We evaluated scale robustness by resizing the SegPC-2021 tests from 300–700 px under subject-exclusive splits, yielding a GAAD-U-Net DSC of 0.88–0.94 (IoU of 0.80–0.92, with +7% overall gain) and proving its efficacy across resolutions. Performance peaks at 700 px (DSC = 0.94, IoU = 0.92) and dipped 6% at 300 px (DSC = 0.88, IoU = 0.80), retaining 94% at 500 px+ (Figure A1). The results indicate that the model is robust to downscaling yet leverages high-resolution detail for improved performance.

To probe stain and intensity robustness, we jittered on a random WBC image from the Cropped SegPC-2012 dataset (±20% contrast, ±30% brightness, +15% over-stain, +25% saturation). The high brightness peaked at a DSC of 0.941 and IoU of 0.892 (+1% gain), while the low brightness dipped to a DSC of 0.900 and IoU of 0.825 (4% drop). Overall, GAAD-U-Net maintained a DSC term over 0.9, proving its robustness under synthetic color jitter and contrast changes. Figure A2 underscores the clinical deployment strength.

## Appendix G. Gating Mechanism Evaluation

To validate the selectivity of the gating mechanism, we conducted an ablation study on our architecture, evaluating its effect on the true positive (TP) and false positive (FP) rates. In this context, a reduction in *TP*s signifies the suppression of valid WBC regions, whereas a decrease in *FP*s indicates the filtering of irrelevant background areas. The results are presented alongside the prediction masks in Figure A3.

## Appendix H. Fail Cases

Figure A4 illustrates sample cases wherein the GAAD-U-Net architecture exhibited suboptimal performance for WBCs, owing to overlapping structures. This limitation arises from the inherent constraints of semantic segmentation paradigms, which struggle to delineate individual instances within crowded regions. To mitigate this challenge, an instance segmentation framework could be employed to enhance discriminatory capabilities. Figure A5 depicts WBC images subjected to staining artifacts, wherein our model encountered difficulties attributable to the irregular patterns prevalent in the affected areas. Furthermore, Figure A6 highlights instances of segmentation failure for the cytoplasm class, primarily due to the ambiguous boundaries shared with the background. These latter challenges—namely handling staining variations and resolving boundary ambiguities—may be effectively addressed through domain adaptation techniques, thereby conferring greater robustness to the overall system.
